# Suicide behaviour after hospitalisation and related factors: a case report.

**DOI:** 10.1192/j.eurpsy.2023.2371

**Published:** 2023-07-19

**Authors:** P. Setién Preciados, A. Sanz Giancola, E. Arroyo Sánchez, M. Martín Velasco, I. Romero Gerechter

**Affiliations:** 1 Psychyatry, Hospital Universitario Príncipe de Asturias, Alcalá de Henares, Spain

## Abstract

**Introduction:**

Suicide is a global epidemic, with the World Health Organization (WHO) estimating that there are roughly 800,000 suicides annually, accounting for 1.4% of all deaths, and making suicide the 18th leading cause of death in 2016 (World Health Organization. There is a pressing need to better understand factors that contribute to suicide risk. One important domain for suicide prevention is inpatient psychiatric treatment, as many patients are admitted precisely in order to reduce their risk of suicide. Although inpatient psychiatric treatment is often used for suicide risk prevention the risk of suicide after inpatient treatment remains high. Patients who have been recently discharged have a greater risk of suicide than non-hospitalised mentally ill people.

**Objectives:**

Review suicidal risk after hospitalisations and the factors that may have an influence on it.

**Methods:**

Presentation of a patient’s case and review of existing literature, in regards to the rate of suicide after a patient is released from psychiatric hospitalisation and the factors that surround it.

**Results:**

The patient in question is admitted into a psychiatric ward with a diagnosis of severe psychotic depression, after a suicide attempt trying to dissect his arms’ blood vessels. Health professionals at the hospital attend to his needs and the patient sees improvement. Not long after his release, there is a second hospital admission, which doesn’t have the same result and after his release he successfully ends his life. What comes to mind with these sorts of patients is: what kind of help would they have needed? Why hospital admission was not enough? And which factors and profile of patient is more prone to develop suicide behaviour?

**Conclusions:**

Admissions at psychiatric wards always have to be thought of as a beneficial resource for patients. There are some cases in which patients do not get the help they need by being hospitalised, increasing the risk of comitting suicide. A lot more studies will have to be carried out to understand what variables play a part in this. Meanwhile an improvement in outpatient care to support patients after hospital release is crucial.

**Disclosure of Interest:**

None Declared

